# Evaluation of Ultrasonic Stress Wave Transmission in Cylindrical Roller Bearings for Acoustic Emission Condition Monitoring

**DOI:** 10.3390/s22041500

**Published:** 2022-02-16

**Authors:** Bart Scheeren, Miroslaw Lech Kaminski, Lotfollah Pahlavan

**Affiliations:** Department of Maritime and Transport Technology, Delft University of Technology, 2628 CD Delft, The Netherlands; m.l.kaminski@tudelft.nl (M.L.K.); l.pahlavan@tudelft.nl (L.P.)

**Keywords:** acoustic emission, low-speed roller bearings, structural health monitoring, elastic stress waves

## Abstract

In the condition monitoring of bearings using acoustic emission (AE), the restriction to solely instrument one of the two rings is generally considered a limitation for detecting signals originating from defects on the opposing non-instrumented ring or its interface with the rollers due to the signal energy loss. This paper presents an approach to evaluate transmission in low-speed roller bearings for application in passive ultrasound monitoring. An analytical framework to describe the propagation and transmission of ultrasonic waves through the geometry and interfaces of a bearing is presented. This framework has been used to evaluate the transmission of simulated damage signals in an experiment with a static bearing. The results suggest that low- to mid-frequency signals (<200 kHz), when passing through the rollers and their interfaces from one raceway to the other, can retain enough energy to be potentially detected. An average transmission loss in the range of 10–15 dB per interface was experimentally observed.

## 1. Introduction

Highly loaded low-speed roller bearings typically form crucial connections in offshore installations. Notable examples of these are the slewing and sheave bearings in offshore heavy-lifting cranes. As these structures are held to the highest standards of safety and reliability due to the remote nature of the offshore environment, assurance of the integrity of those critical roller bearings is of special concern. Conventional methods for the condition monitoring of roller bearings, i.e., vibration monitoring and strain monitoring [1,2], are known to perform poorly in low-speed applications. Alternative methods, such as lubrication analysis [3] and electrostatic monitoring [4], temperature monitoring [5], and ultrasonic techniques have been proposed and explored as well. An elaborate review of these techniques has been provided by Lu et al. [6], El-Thalji and Jantunen [7], de Azevedo et al. [8], and Rai and Upadhyay [9]. For its outstanding potential to detect early-stage degradation, e.g., sub-surface cracking, passive ultrasonic monitoring, also commonly referred to as acoustic emission (AE) monitoring, is further investigated in this paper.

AE is based on the principle that the development of degradation, e.g., crack growth, is associated with the release of elastic energy. Various applications of AE for monitoring damage in different fields have been successfully reported so far, e.g., for steel bridges [10,11,12], concrete structures [13,14,15,16], and composite laminates [17,18,19]. AE signals propagate through the material in the form of elastic stress waves. In roller bearings, these waves radiate from the source event inside the bearing, propagate through the raceways, are transmitted through the interfaces between the rollers and raceways, and also partly through the lubricant. They are reflected, scattered, and diffracted, and finally, if the waves retain sufficient energy, they can be detected and measured on a surface that is accessible for instrumentation.

One of the first applications of AE for bearing condition monitoring has been described by Balderston [20], who successfully demonstrated the potential for detecting defects prior to failure in a laboratory environment. A decade later, Rogers pioneered the application of the technique on an asset in the field [21]—specifically on the slewing bearing of an offshore crane. In his paper, he describes one of the primary challenges of interpreting field measurements; detecting no damage when there most likely is none, which defines the crucial intersection between false alarms and missed detections. In the following years, several more studies investigated the applicability of AE for bearing condition monitoring [22,23,24], typically presenting it as an alternative to vibration monitoring—particularly for high-noise environments and low-speed applications. Additionally, careful claims are made on the potential of AE to serve as an early warning system, based on the probable detection of early stage subsurface cracking in run-to-failure experiments [25], while also mitigating the uncertainties related to varying operational conditions through probabilistic modelling [26]. Broadly formulated, the state of the art focuses on improved robustness for damage detection [27,28,29], and the estimation of both damage severity [30,31,32,33] and location [34]. For the latter, machine learning techniques are also employed, such as discrete hidden Markov models [35], neural networks [36], and deep learning [37]. These studies show promising first results for gauging the defect size using parameters such as the duration of or the time between AE bursts and demonstrate the potential for defect location identification through the classification of wavelet transformed signals; however, these successes are tempered by their inclusion of solely artificial defects.

In the context of the envisioned application, a distinction must be made between the two regimes of AE bearing condition monitoring. At high rotational speeds, the emission of stress waves is dominated by impact events between the rollers and raceways, and the disturbances caused herein by surface damages or lubrication defects. These are typically of a lower frequency and are referred to as pseudo acoustic emission sources. At low speeds, these impact events become much less significant; thus, what primarily remains are the higher-frequency stress waves generated by microstructure changes in the material. These are true acoustic emission sources.

Within the spectrum of tribology, anything operating below 600 rpm is generally considered to be low-speed [38]; however, for the purpose of bearing condition monitoring, this differentiation is too broad and generic. For rotational speeds between 200–500 rpm, He et al. [39] have shown that speed may greatly influence the quality of AE parameters. For much lower speeds, Miettinen and Pataniitty [40] have shown that the detectability of AE signals decreases with decreasing speeds in the range of 0.5–5 rpm. However, at similar very-low rotational speeds, successes are also obtained through classification approaches by autoregressive coefficients, which have shown to effectively separate noise from damage-induced signals of both natural [41] and artificial origin [42].

Besides rotational speed, the accessibility of the bearing rings is also a challenge faced by many researchers. As such, most of the studies referenced before limited their instrumentation to one of the two rings. A review by Sikorska and Mba [43] identified that, for single ring instrumentation, the detection of defects on the opposing ring is more difficult due to the dynamic transmission path between defect and sensor. However, this transmission mechanism is yet to be properly characterised.

In the present paper, an analytical framework to describe the transmission and propagation of ultrasonic waves through the geometry and interfaces of a roller bearing is introduced. Using this framework, stress wave transmission in a sheave bearing—from one raceway through the rollers to the other raceway—is experimentally investigated, making use of simulated sources. Transmission coefficients are extracted from time–frequency representations of the recorded waveforms. Tests are performed with a static bearing, as the dynamic state of the considered bearings (<20 rpm) is quasi-static from the perspective of stress wave propagation. Through these results, the feasibility of passive ultrasonic condition monitoring of low-speed roller bearings by single ring instrumentation is quantitatively evaluated.

## 2. Methodology

The detectability of developing degradation on different components of a bearing is governed by the transmission of elastic stress waves across the interfaces and through the components of that bearing. To describe and quantify this transmission, a framework is proposed to analyse the propagation of elastic stress waves in complex geometries. This framework approaches propagation, transmission, and detection of a signal as a series of transformations applied to the emitted source signal.

**Figure 1:**
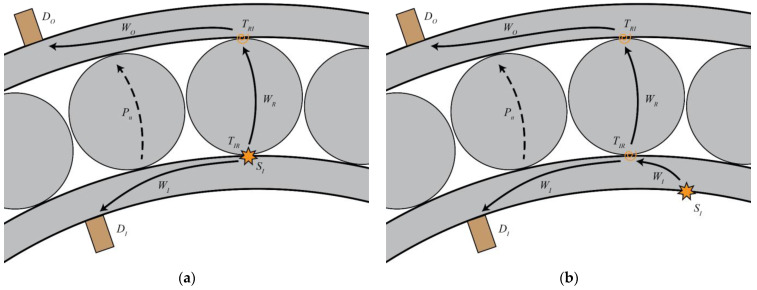
Representation of the wave propagation paths, showing: (**a**) the propagation paths associated with a natural sub-surface defect originating on the inner raceway, and (**b**) the propagation paths as simulated in the experiment. In both, the primary path is represented by a continuous line, and an alternative path as a dashed line.

Limited by the geometry of an assembled bearing, the simulated stress waves cannot be generated at internal locations, where degradation would occur in practice (illustrated in Figure 1a). Instead, the proposed framework allows for the extraction of the transmission coefficients using simulated sources on the external surfaces of the bearing (illustrated in Figure 1b). These coefficients are expected to provide a conservative estimate of the feasibility.

For stress waves propagating directly from the source on the inner raceway to the receiver on that same inner raceway, the transmitted signal may be described in the frequency domain as

(1) $$P_{II}(s_{RI},s_{SI},\omega )=D_{I}(\omega )W_{I}(s_{RI},s_{CI},\omega )W_{I}(s_{CI},s_{SI},\omega )S_{I}(\omega )+P_{N},$$

wherein $P_{II}$ represents the response recorded at the receiver located on the inner raceway of the source emitted on the inner raceway, $D_{I}$ is the coupling transfer function of the receiver on the inner raceway, $W_{I}$ is the wave propagation function of the inner raceway, $S_{I}$ is the source function on the inner raceway, and $P_{N}$ is all the neglected paths, mode conversions, the scattering of the transmitted responses and the background noise. Furthermore, s denotes a spatial location, e.g., the position of a receiver on the inner raceway ($s_{RI}$), a source on the inner raceway ($s_{SI}$), and the contact point between the roller and inner raceway ($s_{CI}$). Finally, ω denotes the angular frequency.

In this formulation, the wave propagation inside of the raceway is split into two contributions, apart from the source to the roller-raceway contact spot $s_{CI}$, and the continuation from that spot to the receiver. It is assumed that propagation along this path is nearly identical to direct propagation between source and receiver, i.e.,

(2) $$W_{I}(s_{RI},s_{SI})\approx W_{I}(s_{RI},s_{CI})W_{I}(s_{CI},s_{SI}).$$

Stress waves propagating from the same source on the inner raceway to a receiver on the opposing outer raceway may be described by

(3) $$P_{OI}(s_{RO},s_{SI},F,\omega )=D_{O}(\omega )W_{O}(s_{RO},s_{CO},\omega )T_{RO}(F,\omega )W_{R}(s_{CO},s_{CI},\omega )T_{IR}(F,\omega )W_{I}(s_{CI},s_{SI},\omega )S_{I}(\omega )+P_{N},$$

wherein $P_{OI}$ represents the response recorded at the receiver located on the outer raceway of the source emitted on the inner raceway, $D_{O}$ *is* the coupling transfer function of the receiver on the outer raceway, $T_{IR}$, and $T_{RO}$ are the transmission functions for the interfaces between the inner raceway and roller, and the roller and outer raceway respectively, and $W_{R}$, and $W_{O}$ are the wave propagation functions of the roller, and outer raceway respectively. Furthermore, additional points in space are defined, which denote the position of a receiver on the outer raceway ($s_{RO}$), and the contact point between the roller and outer raceway ($s_{CO}$). Finally, F denotes the contact force between the roller and raceways.

This formulation uses a similar structure to Equation (1) for describing wave propagation from the source to the contact point and from the contact point to the receiver. The major difference in Equation (3) with respect to Equation (1) is the inclusion of the transmission into, propagation over, and transmission out of the roller. Using the similarity between Equations (1) and (3) a formulation may be established that expresses the recording on the outer raceway as a transformation of the recording on the inner raceway. For this, it is assumed that the receivers have similar transfer functions, and that coupling variation is either negligible or accounted for in pre-processing, such that $D_{O}\approx D_{I}$. Additionally, propagation in the raceways is assumed to be similar, i.e., $W_{I}(s_{RI},s_{CI})\approx W_{O}(s_{RO},s_{CO})$. Then transmission from one raceway to the other is given by

(4) $$P_{OI}(s_{RO},s_{SI},F,\omega )=\underset{A_{R}}{\underbrace{T_{RO}(F,\omega )W_{R}(s_{CO},s_{CI},\omega )T_{IR}(F,\omega )}}P_{II}(s_{RI},s_{SI},\omega ).$$

It shows that the relative amplitude $A_{R}$ is composed of the transmission functions $T_{IR}$ and $T_{RO}$, and propagation through the roller is $W_{R}$.

If the attenuation of waves in the roller is considered to be reasonably small due to the relatively short propagation path with respect to the wavelength ($\left|W_{R}(s_{CO},s_{CI})\right|\approx 1$), under the assumption of negligible phase shift, and transmission over the interfaces is assumed to be independent of the propagation direction ($T_{RO}\approx T_{IR}$), then $A_{R}$ represents about twice the value for transmission over a single interface in the dB scale.

### 2.1. Experimental Set-Up

**Figure 2:**
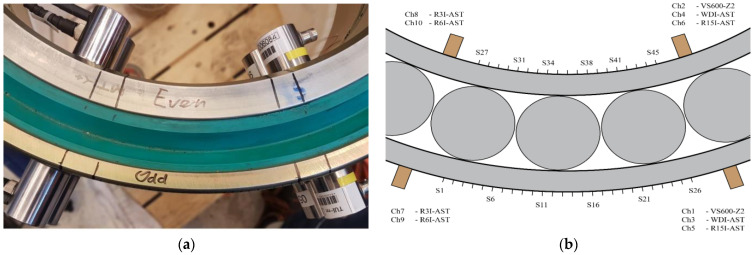
The top view of the experimental set-up, showing: (**a**) a picture of the instrumented bearing, and (**b**) a to-scale illustration indicating the sensor and source positions. Note that the roller positions in the illustration are purely indicative, and unknown in the actual experiment.

A cylindrical roller bearing of type F-566437.NNT by MTK Bearings was instrumented on the exterior of the inner and outer ring with standard piezoelectric acoustic emission sensors. The bearing had been in service in a sheave of a 4000 mT heavy-lifting crane on a deep-water construction vessel. During the tests, the bearing was positioned horizontally to omit self-loading with no external loads applied. These conditions were used to obtain a conservative estimate of the worst transfer characteristics. A picture and an illustration of the set-up are shown in Figure 2

The utilised type F-566437.NNT of MTK Bearings is a double row full complement cylindrical roller bearing. It has an outer diameter of 500 mm, an inner diameter of 360 mm, and a width of 175 mm. The inner ring is split in two segments of half width. Both inner rings and the outer ring act as raceways. The two rows each contain a compliment of 30 rollers with a diameter of 44.8 mm and a width of 55 mm. The rings and raceways are all made out of GCr15 bearing steel. It has the respective static and dynamic load ratings of 6200 kN and 2800 kN.

A bearing segment of 30 degrees had been selected for source excitation to guarantee the presence of at least two rollers within the segment. Pencil lead breaks were performed in 45 regularly spaced source locations on the inner and outer raceway to excite stress waves. In accordance with ASTM-E976 [44], 10 Hsu-Nielsen excitations were performed at each 5 mm spaced source location using 0.5 mm 2H pencil leads.

Transmission of the excited waves through the bearing components was recorded in the frequency range of 30–650 kHz. For this purpose, five types of commercial piezoelectric AE sensor were used, i.e., (i) 30 kHz resonant R3I-AST, (ii) 60 kHz resonant R6I-AST, (iii) 150 kHz resonant R15I-AST, (iv) wideband WDI-AST, and (v) 600 kHz resonant VS600-Z2. The first four (i–iv) were sourced from Physical Acoustics Corporation (Princeton Jct., NJ, USA) and contain an integral pre-amplifier with a gain of 40 dB. The fifth (v) was sourced from Vallen Systeme (Wolfratshausen, Germany) and was amplified using an external AEP5H pre-amplifier set to the same gain of 40 dB. A Vallen Systeme AMSY-6 system, outfitted with ASIP-2/A signal processing cards, was used to sample the detected waveforms with 40 MHz for both feature- and transient-data whenever a 40 dB threshold was crossed. This sample included a 200 µs pre-trigger recording before the threshold crossing. No additional digital band-pass filters were applied prior to storing the waveforms.

### 2.2. Wavelet Transform

In order to estimate the frequency-dependent characteristics of wave propagation of roller bearings, the recorded time-series were converted to the time–frequency domain using the wavelet transform. The procedure for this transform can be described as a series of cross-correlations with rescaled and translated versions of a reference signal, i.e., the wavelet function. In the time domain this transformation is defined as

(5) $$w_{\psi }(\tau ,s)\equiv \int_{-\infty }^{\infty }\frac{1}{s}\psi^{*}\left(\frac{t-\tau }{s}\right)x(t)dt,$$

wherein $w_{\psi }$ represents the wavelet transform of signal x(t) with wavelet function ψ(t), and $\psi^{*}$ denotes the complex conjugate of the wavelet ψ. Furthermore, τ denotes the translation of the wavelet function in time, and s the scale of the wavelet. In this study a Morse wavelet is used [45,46,47,48], which is defined in the frequency domain. The time domain representation of this wavelet is obtained using the inverse Fourier transform

(6) $$\psi_{\beta ,\gamma }(t)=\frac{1}{2\pi }\int_{-\infty }^{\infty }\Psi_{\beta ,\gamma }e^{i\omega t}d\omega ,$$

with the Morse wavelet defined as

(7) $$\Psi_{\beta ,\gamma }(\omega )\equiv a_{\beta ,\gamma }\omega^{\beta }e^{-\omega^{\gamma }}\times \left\{ \begin{array}{l} 1\;\;\;\;\;\omega >0\\ \frac{1}{2}\;\;\;\;\omega =0\\ 0\;\;\;\;\omega <0 \end{array}\right.,$$

wherein

(8) $$a_{\beta ,\gamma }\equiv 2{\left(\frac{e\gamma }{\beta }\right)}^{\beta /\gamma }.$$

In Equations (6), (7) and (8), $\Psi_{\beta ,\gamma }$ and $\psi_{\beta ,\gamma }$ denote the respective frequency and time domain representations of the Morse wavelet, with $\alpha_{\beta ,\gamma }$ representing a normalising constant. Furthermore, e is Euler’s number, and β and γ denote the respective order and family of the wavelet, which control the low-frequency behaviour and high-frequency decay.

This procedure transforms the time domain signal into the translation-scale domain, which is synonymous with the time–frequency domain. To express the former in the latter, one must first consider that translation space is representative of the temporal space, such that $t_{\tau }=\tau$. Then, to express scale in terms of frequency, one must consider that three meaningful frequencies may be defined for a wavelet; (i) the peak frequency, where the wavelet amplitude is maximised, (ii) the energy frequency, where the centroid of the wavelet energy is situated, and (iii) the instantaneous frequency evaluated at the centre of the wavelet, where t=0.

Since each of these frequencies is considered to be a valid interpretation of the frequency of the wavelet, a definite mapping of scale to frequency can only be obtained if these three frequencies coincide. This is nearly the case for a Morse wavelet with γ=3 [45]. As this is the family of Morse wavelet used in this study, the wavelet frequency is approximated using the peak frequency, which is given by

(9) $$\omega_{\beta ,\gamma }\equiv {\left(\frac{\beta }{\gamma }\right)}^{1/\gamma },$$

in which $\omega_{\beta ,\gamma }$ denotes the peak frequency. Subsequently, the scale frequency is linked to the peak frequency through the scale as

(10) $$\omega_{s}=\frac{\omega_{\beta ,\gamma }}{s},$$

wherein $\omega_{s}$ denotes the scale frequency.

For each sensor type a frequency window was selected for which Equation (4) was evaluated. Making reference to the same numbering as used before for the sensor types, these windows were (i) 30–55 kHz, (ii) 55–80 kHz, (iii) 80–180 kHz, (iv) 180–550 kHz, and (v) 550–650 kHz. For each sensor pair on the opposing raceways, the experimental relative amplitude was determined as the difference between the logarithmic peak value of the first arrival of the windowed wavelet transformed signals.

### 2.3. Coupling Variation

In pre-processing, a procedure for coupling variation correction was implemented to compensate for possible differences in the coupling between receiver pairs (as discussed with the assumptions under which Equation (4) holds). For each receiver pair, assuming that the coupling variation predominantly manifest itself in signal amplification variation (and not phase), a scaling factor was applied to the signals recorded on the inner raceway.

The scaling factors were determined by comparing direct recordings at fixed distances from each sensor on the outer raceway, to the corresponding direct recordings on the inner raceway. The mean difference in logarithmic peak amplitudes of the frequency-windowed time–frequency representation of the signals determined the correction factor.

Since only the relative amplitudes for each receiver pair were used throughout the analysis, the signal amplitudes did not need to be corrected individually to obtain absolute values.

### 2.4. Overview of Processing Approach

**Figure 3:**
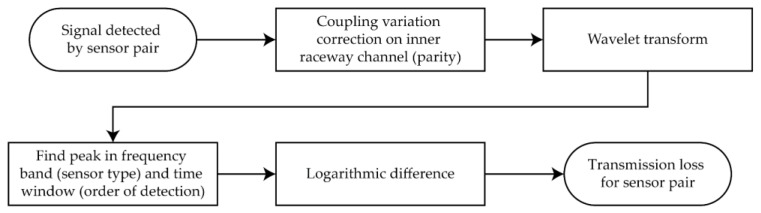
Overview of procedure for data processing.

An overview of the full processing procedure is included in Figure 3

## 3. Results and Discussion

**Figure 4:**
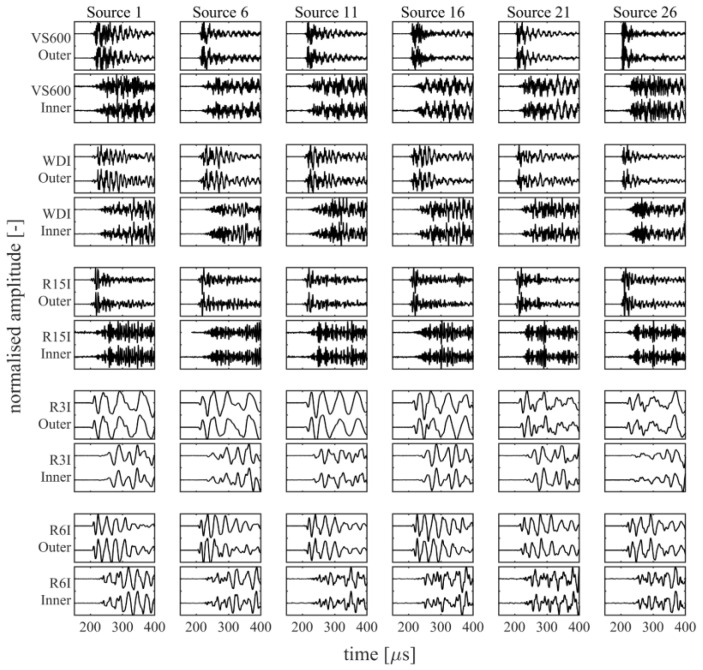
Overview of the recorded waveforms for several outer raceway sources. Each block depicts a 250 µs sample of two waveforms that have been normalised with their respective peak amplitudes.

**Figure 5:**
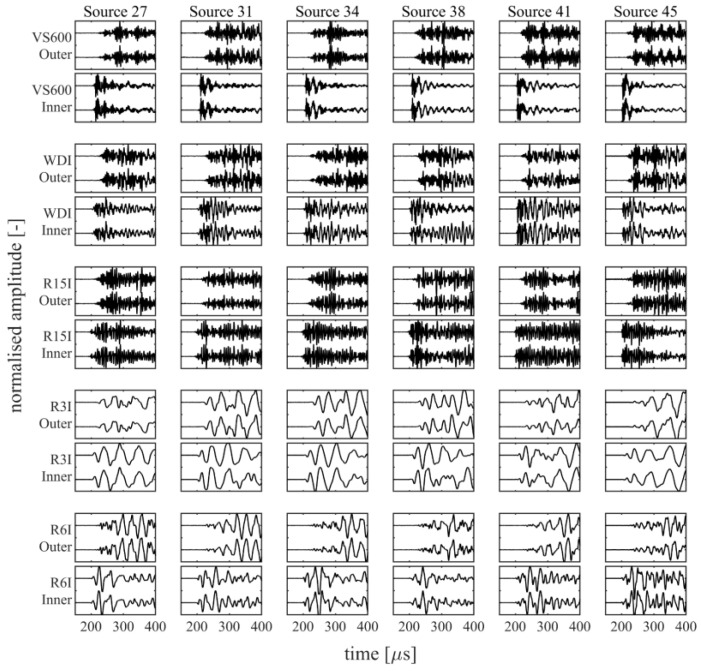
Overview of the recorded waveforms for several inner raceway sources. Each block depicts a 250 µs sample of two waveforms that have been normalised with their respective peak amplitudes.

For the combined 45 source locations, a total of 475 events were recorded that surpassed the minimal requirement of being detected by at least three of the five sensor pairs, each with a threshold of 40 dB. Out of these 475 events, 419 contained recordings for all channels. The events that were not recorded by all channels typically originated from an outer raceway source and missed a recording for one of the R15I-AST sensors, due to an insufficiently low threshold for some of the weaker source excitations. An overview of several recorded waveforms for a select few source locations is presented in Figure 4 for the outer raceway sources and Figure 5 for the inner raceway sources.

Time–frequency representations of the recorded waveforms were obtained through the continuous wavelet transform. Herein, the used wavelet was a Morse wavelet with γ=3 (family) and β=20 (order). The family was selected to obtain an unambiguous wavelet frequency, whereas the order was tuned to obtain a sufficiently narrow frequency localisation, while retaining ample time localisation. Slight adjustments to these empirically tuned values were not expected to influence the results of this study.

**Figure 6:**
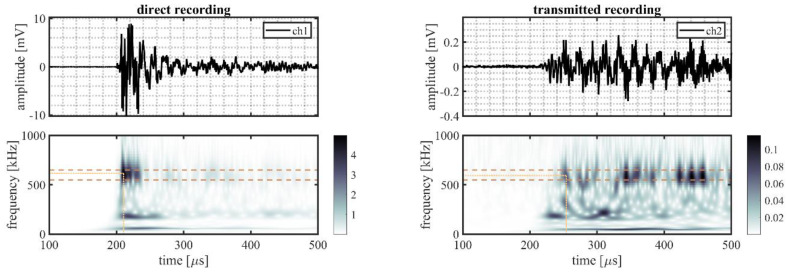
Randomly selected event recorded by VS600-Z2 sensors, originating from a source location S22, showing: the time traces recorded on the outer raceway (ch1, **top left**) and on the inner raceway (ch2, **top right**), and the time–frequency representation of the recorded signals on the outer (**bottom left**) and the inner (**bottom right**) raceways. The detected magnitude in the time–frequency representations of the signals are indicated by the intersection of the dotted lines; the dashed lines indicate the frequency range of interest (550–650 kHz).

**Figure 7:**
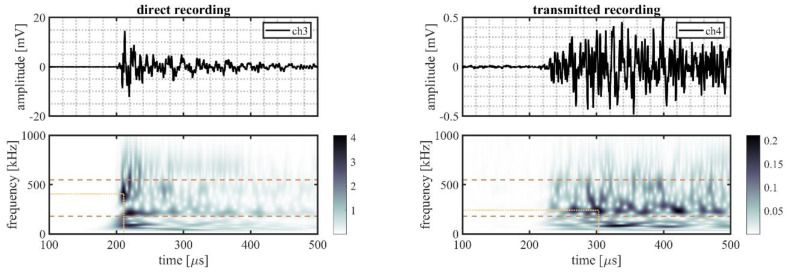
Randomly selected event recorded by WDI-AST sensors, originating from a source location S22, showing: the time traces recorded on the outer raceway (ch3, **top left**) and on the inner raceway (ch4, **top right**), and the time–frequency representation of the recorded signals on the outer (**bottom left**) and the inner (**bottom right**) raceways. The detected magnitude in the time–frequency representations of the signals is indicated by the intersection of the dotted lines; the dashed lines indicate the frequency range of interest (180–550 kHz).

**Figure 8:**
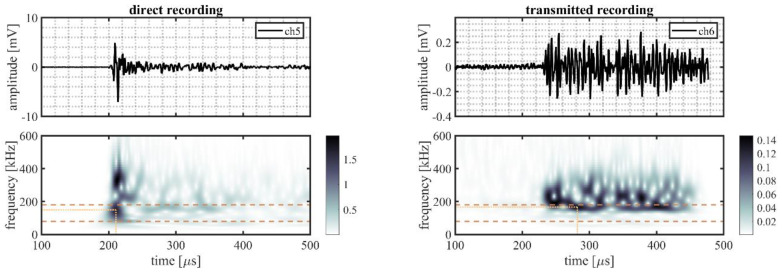
Randomly selected event recorded by R15I-AST sensors, originating from a source location S22, showing the time traces recorded on the outer raceway (ch5, **top left**) and on the inner raceway (ch6, **top right**), and the time–frequency representation of the recorded signals on the outer (**bottom left**) and the inner (**bottom right**) raceways. The detected magnitude in the time–frequency representations of the signals is indicated by the intersection of the dotted lines; the dashed lines indicate the frequency range of interest (80–180 kHz).

**Figure 9:**
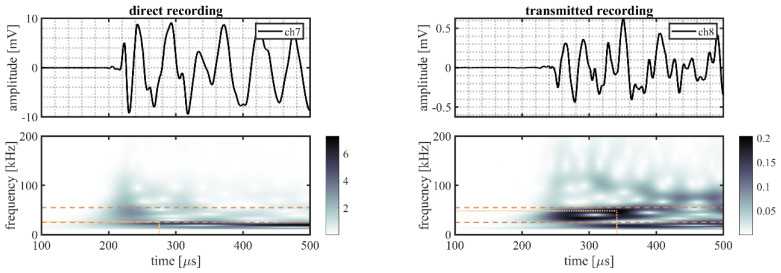
Randomly selected event recorded by R3I-AST sensors, originating from a source location S22, showing the time traces recorded on the outer raceway (ch7, **top left**) and on the inner raceway (ch8, **top right**), and the time–frequency representation of the recorded signals on the outer (**bottom left**) and the inner (**bottom right**) raceways. The detected magnitude in the time–frequency representations of the signals is indicated by the intersection of the dotted lines; the dashed lines indicate the frequency range of interest (30–60 kHz).

**Figure 10:**
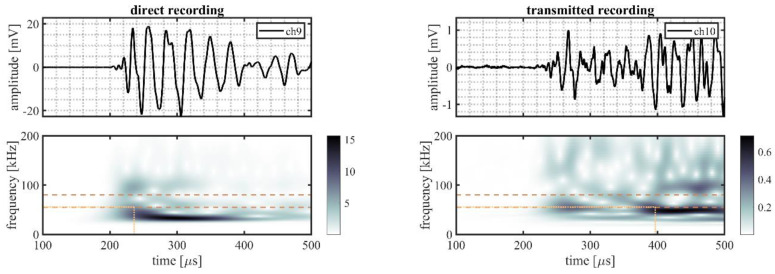
Randomly selected event recorded by R6I-AST sensors, originating from a source location S22, showing the time traces recorded on the outer raceway (ch9, **top left**) and on the inner raceway (ch10, **top right**), and the time–frequency representation of the recorded signals on the outer (**bottom left**) and the inner (**bottom right**) raceways. The detected magnitude in the time–frequency representations of the signals are indicated by the intersection of the dotted lines; the dashed lines indicate the frequency range of interest (55–80 kHz).

A randomly selected event is illustrated in more detail to better explain the processing procedure. This event originated from source location S22 on the outer ring (indicated in Figure 2b by the mark directly right of S21). The time traces and time–frequency representations for all five sensor pairs of this randomly selected event are shown in Figure 6 (VS600-Z1), Figure 7 (WDI-AST), Figure 8 (R15I-AST), Figure 9 (R3I-AST), and Figure 10 (R6I-AST). For the rest of this section when the subfigures are mentioned, it is meant to refer back to those figures collectively.

Initial observations of the time traces in the top subfigures show the odd channels to detect the signal earlier and with higher peak-amplitude. These observations indicate that the source signal originated from the same ring the odd numbered channels were located on, i.e., the outer raceway. As such, this event evaluated the transmission from the outer raceway to the inner raceway. It must be noted that this propagation direction was opposing the transmission explicitly denoted in Equation (4).

For the time–frequency representations of the signal recorded on the source side, shown in the bottom left subfigure, the peak magnitude of the wavelet transform within the considered frequency range was selected as the reference magnitude of the source signal. This peak is indicated by the intersection of the dotted lines.

On the opposing raceway, shown in the bottom right subfigure, the reference magnitude for the transmitted signal was obtained by selecting the peak magnitude within the considered frequency band that occurred in the range of 50–250 µs following on the selected reference magnitude of the source signal. This selection was made to avoid picking a maximum resulting from interacting reflections. This approach, however, is not fault proof, as it may be susceptible to interacting arrivals from different transfer paths of similar length. This effect is partly mitigated by averaging over multiple source locations.

From the two selected reference magnitudes, the relative amplitude of the transmitted signal was obtained by determining the difference between their logarithmic-scale values.

A noteworthy observation may be that the detection of the peak magnitudes for the WDI-AST type sensors, as shown in Figure 7, occurred at the lower end of the selected frequency range. This may place the results for this sensor type closer to the mid-frequency range, than the high-frequency range.

**Figure 11:**
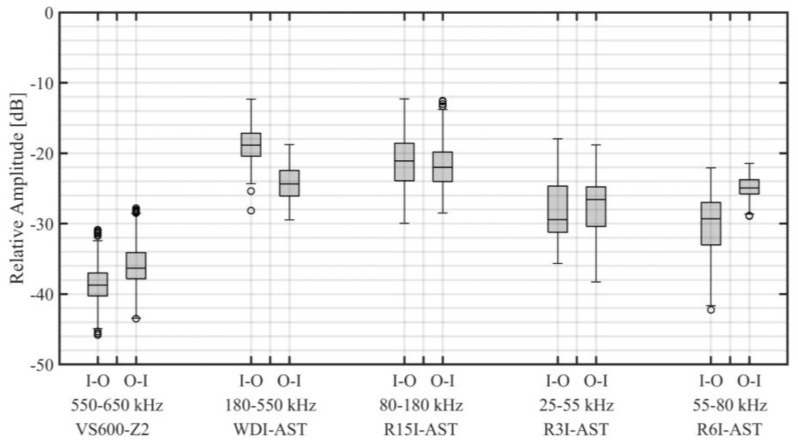
Overview of relative amplitude for different frequency ranges and transmission directions, where I-O refers to transmission from the inner ring to the outer ring, and O-I to transmission from the outer ring to the inner ring. The shaded box denotes the median and the range between the lower and the upper quartile, the circles represent outliers, and the whiskers indicate the non-outlier minima and maxima.

For each source excitation, on each source location, the same procedure was applied for each sensor type. An overview of all relative amplitudes is shown in Figure 11. In this figure the relative amplitude for each sensor type and transmission direction is depicted as an individual boxplot. The signal transmission from the outer to the inner raceway, i.e., the direction of the selected example event, is indicated by the label O-I, and presented on the right side for each sensor group. The signal transmission from the inner to the outer raceway, i.e., the direction for which Equations (1), (3) and (4) was established, is indicated by the label I-O, and presented on the left side for each sensor group.

The results indicate that (i) transmission was most favourable in the range of 80–180 kHz (as recorded by R15I-AST), (ii) for this frequency range, a mean amplitude drop of 20–25 dB is to be expected, (iii) the general performance of WDI-AST resembles R15I-AST, indicating consistent near mid-frequency selection from the broadband window, (iv) for lower frequencies, mean transmission losses were slightly higher, ranging between 25–30 dB, and (v) higher frequencies suffered from significant mean transmission losses (>35 dB).

Increased transmission losses at higher frequencies were expected to be partly related to non-negligible attenuation in the roller. For a frequency of 600 kHz the wavelength was notably smaller than the diameter of the roller, which could give rise to spreading and increased attenuation. Mid- and low-frequency results show that the assumption of negligible attenuation was reasonable for this geometry below 200 kHz, representing wavelengths in the same order of magnitude as the diameter of the roller.

Note that the presented transmission losses are in terms of the relative amplitude $A_{R}$ as defined by Equation (4), and thus include transmission into, through, and out of the roller. Considering the aforementioned assumptions of negligible attenuation inside rollers and directional independence, it may generally be stated the transmission loss for a single interface at mid-frequency was in the order of 10–13 dB.

Moreover, the presented relative amplitude was obtained for a bearing in horizontal position; thus, without applied loading or self-weight. It is assumed that this position provides a conservative estimate on transmission, when compared to a loaded bearing in operation.

## 4. Conclusions

A framework to describe and assess the transmission of ultrasonic waves through the geometry and interfaces of a roller bearing is presented. Combined with experimental data, this framework was used to quantify stress wave transmission from one raceway, through a roller, to the other raceway. The experiments made use of a statistically large sample of Hsu-Nielsen sources, and multiple AE sensors to cover a frequency range from 30 to 650 kHz. Quantitative analysis of the recorded signals suggests that (i) the transmission of AE signals through rollers and their interfaces was potentially of sufficient strength to be recorded and distinguished from the background noise, (ii) transmission was most favourable in the mid-frequency range (80–180 kHz), (iii) for mid frequencies, a transmission loss of 10–13 dB is to be expected per interface, (iv) for lower frequencies, transmission was slightly less favourable (12–15 dB per interface), and (v) for higher frequencies, transmission degraded significantly (17–20 dB per interface).

The current study demonstrates the principal feasibility of single ring instrumentation in bearing condition monitoring, when rotation may be considered quasi-static relative to stress wave propagation. However, since only static tests were performed, the influences of dynamic effects on the ultrasonic noise are yet to be assessed and quantified in future work.

## Data Availability

The data presented in this study are available upon request from the corresponding author.
